# Where backyard poultry raisers seek care for sick poultry: implications for avian influenza prevention in Bangladesh

**DOI:** 10.1186/s12889-018-5819-5

**Published:** 2018-08-03

**Authors:** Nadia Ali Rimi, Rebeca Sultana, Kazi Ishtiak-Ahmed, Najmul Haider, Eduardo Azziz-Baumgartner, Nazmun Nahar, Stephen P. Luby

**Affiliations:** 1 Program for Emerging Infections (PEI), Infectious Diseases Division (IDD), icddr, b, 68, Shaheed Tajuddin Ahmed Sharani, Mohakhali, Dhaka, 1212 Bangladesh; 2 Department of Public Health, University of Copenhagen, Copenhagen, Denmark; 3 University of Copenhagen, Copenhagen, Denmark; 4 Technical University of Denmark, Copenhagen, Denmark; 5 Centers for Disease Control and Prevention (CDC), Atlanta, GA USA; 6 Stanford University, Stanford, California, USA

**Keywords:** Backyard poultry raiser, Informal care provider, poultry care provider, poultry disease, Avian influenza, Perception, Bangladesh

## Abstract

**Background:**

In Bangladesh, backyard poultry raisers lack awareness of avian influenza and infrequently follow government recommendations for its prevention. Identifying where poultry raisers seek care for their ill poultry might help the government better plan how to disseminate avian influenza prevention and control recommendations.

**Methods:**

In order to identify where backyard poultry raisers seek care for their ill poultry, we conducted in-depth and informal interviews: 70 with backyard poultry raisers and six with local poultry healthcare providers in two villages, and five with government veterinary professionals at the sub-district and union levels in two districts during June–August 2009.

**Results:**

Most (86% [60/70]) raisers sought care for their backyard poultry locally, 14% used home remedies only and none sought care from government veterinary professionals. The local poultry care providers provided advice and medications (*n* = 6). Four local care providers had shops in the village market where raisers sought healthcare for their poultry and the remaining two visited rural households to provide poultry healthcare services. Five of the six local care providers did not have formal training in veterinary medicine. Local care providers either did not know about avian influenza or considered avian influenza to be a disease common among commercial but not backyard poultry. The government professionals had degrees in veterinary medicine and experience with avian influenza and its prevention. They had their offices at the sub-district or union level and lacked staffing to reach the backyard raisers at the village level.

**Conclusions:**

The local poultry care providers provided front line healthcare to backyard poultry in villages and were a potential source of information for the rural raisers. Integration of these local poultry care providers in the government’s avian influenza control programs is a potentially useful approach to increase poultry raisers’ and local poultry care providers’ awareness about avian influenza.

## Background

Bangladesh is a country of more than 150 million people, with 64% living in rural villages [1]. Approximately 71% of the rural households raise backyard poultry [2]. Bangladeshi backyard poultry raisers come into frequent close contact with poultry every day, including touching poultry while putting them into sheds, feeding sick poultry by hand, and slaughtering poultry [3]. Since its first detection in 2007, highly pathogenic avian influenza A (H5N1) virus has become endemic in Bangladesh [4]. Eight human infections of H5N1 virus have been reported since 2008, including a fatal case [5].

In March 2007, the Government of Bangladesh organized a nationwide mass media campaign through radio, television and newspapers, and conducted public meetings through government veterinary officials at sub-district level to disseminate guidelines to prevent avian influenza infection in humans [6]. Following the campaign, a nationwide survey indicated that 49% of backyard poultry raisers recalled hearing about avian influenza during 2007 [2]. A subsequent nationally representative survey among backyard poultry raisers conducted in 2009–2012 reported that only 40% of respondents had recalled hearing about avian influenza or the government prevention guidelines [6]. Television, neighbors, family, and friends were the main sources of information about avian influenza prevention [2, 6, 7]. These studies also identified that Bangladeshi backyard poultry raisers infrequently followed government recommendations for prevention [2, 6, 7] probably because they did not recognize the disease or consider themselves at risk. The fatal case was reported after these studies, which might have contributed to low risk perception. Nevertheless, these studies suggest that existing communication channels were not optimal for reaching backyard poultry raisers or improving backyard raisers’ disease risk perception.

Selecting culturally tailored messages and frequently used local communication networks might help the government improve access to poultry raisers to disseminate avian influenza prevention and control recommendations. In order to improve risk communication to people who raise poultry, this study aimed to identify where backyard poultry raisers sought advice and healthcare for their sick poultry and to explore rural poultry care providers’ knowledge and perceptions about avian influenza.

## Methods

During June through August in 2009, a qualitative research team collected data from one rural village from each of Rajshahi and Chittagong districts [8, 9], the largest and third largest backyard poultry raising area in Bangladesh [10]. Rajshahi in the northwest and Chittagong in the southeast of Bangladesh were chosen to capture practices in two geographically and socio-culturally distinct places of the country. Villages were purposively selected because of their small size, accessibility, and being typical in the region in terms of demographic and geographic characteristics, i.e., agriculture as the main occupation, inhabitant with Muslim majority and located in floodplains. The sites were under surveillance for avian influenza and flocks had yet to test positive for H5N1 virus [11].

To explore where poultry raisers sought advice and healthcare for their sick poultry and the reasons behind their actions, the team conducted informal interviews [12] with backyard poultry raisers about diverse practices until they reached saturation [13], i.e., they repeatedly received similar information from different participants. The team conducted in-depth interviews with all local poultry healthcare providers that the raisers mentioned and all the government veterinary service providers assigned to provide veterinary care in these selected villages. The team explored local and government providers’ knowledge about avian influenza, their role in the treatment of ill backyard poultry and their reasoning behind their practices. For the interviews, the team used unstructured guidelines that included topics to explore and relied on the spontaneous generation of questions during the natural flow of conversation [12]. The team invested substantial time building rapport with the informants in an effort to improve the quality of elicited information. The team recorded the interviews using audio recorders and field notes, then transcribed the recorded data verbatim and expanded the field notes. Then they reviewed the transcriptions and field notes to identify the emerging themes relevant to study objectives. They manually coded data according to themes to identify patterns and analyzed these to prepare a summary of each theme/subtheme [14]. They calculated median and inter-quartile range (IQR) for age and conducted Fisher’s exact test to measure the significance of the difference in years of schooling among different groups.

## Results

### Demographic information

The team interviewed a total of 70 backyard poultry raisers, six local poultry care providers and five government veterinary service providers (Table 1). Most (89%, 62/70) of the poultry raisers were female. The median age of the raisers was 38 years (IQR: 30–45). The majority (73%) of the raisers had less than six years of education. All the local poultry care providers and government veterinary service providers were male. The median age of local poultry care providers was 36 years (IQR: 27–43) and government veterinary service providers was 48 years (IQR: 38–52). Among the six local care providers, three had primary, two had secondary or higher secondary education and one had a Doctor of Veterinary Medicine (DVM) degree. Three of the government service providers had DVM degrees and two were Field Assistants in Artificial Insemination (FAAI) who received training on artificial insemination for cattle from government institutions after passing higher secondary examination.

**Table 1:** Categories and number of informants interviewed in Rajshahi and Chittagong study villages, Bangladesh, 2009

Categories of informants	Rajshahi	Chittagong	Total
Backyard poultry raisers	25	45	70
Local poultry care providers	3	3	6
Government veterinary professionals	3	2	5

### Local poultry care providers and government veterinary care providers

The local poultry care providers gave advice free and sold medicine to raisers with sick poultry; four had medicine shops in the village markets, where one can buy products with or without a prescription, and two conducted household visits. Authors deliberately avoided use of the word ‘pharmacy’ because usually a ‘pharmacy’ is the part of the drug store where pharmacists process the prescriptions. In Bangladesh, there rarely exist provisions for the deliveries of professional pharmacy care practices or services in the medicine shops, where one can buy drugs, aspirin, vitamins, tissues and some other related products. One of the two mobile local poultry care providers was a non-government organization veterinarian, who also worked as a private practitioner in the area. Four of the local poultry care providers provided service for both human and animal illness.

The government had a livestock office and 1–2 registered veterinarians at each sub-district level to provide animal care. The government service providers were available for poultry care only during office hours (9 am–5 pm). In some sub-districts, there were two types of operations at the union level, which is the smallest administrative unit consisting of several villages, an artificial insemination point and a livestock welfare center. In each of our sites, there was only one field staff; this person mainly carried out artificial insemination of cattle and goats within the union. Field staff did not receive a government salary but used government resources (i.e., office space and equipment) to provide service in exchange for remuneration from the cattle owner.

**Fig. 1:**
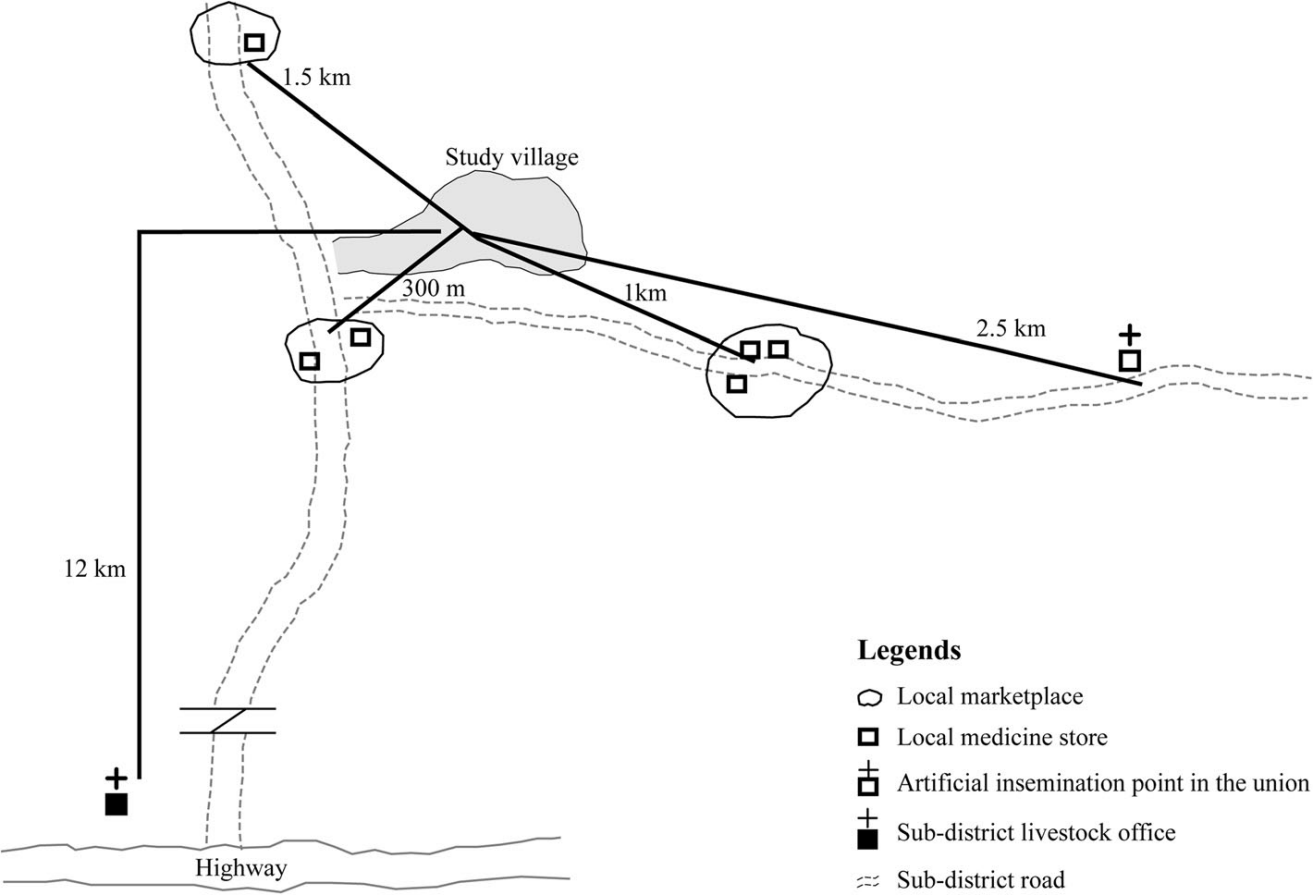
Location of local medicine shops and livestock offices surrounding the Rajshahi study site, Bangladesh, 2009

The local poultry care providers were available outside of office hours and on weekends and were more accessible to the backyard raisers than were the government providers. For example, in the Rajshahi study site, there were six medicine shops in three markets within 300 m to 1.5 km of the study village (Fig. 1) and the local care providers lived inside or near the study village. In contrast, the closest government veterinary service was the artificial insemination point, which was 2.5 km away from the study village; the closest sub-district livestock office was located at a city 12 km away from the study village.

### Backyard poultry raisers’ preference for seeking care for poultry

Most (86%, 60/70) backyard poultry raisers sought care for sick poultry from local poultry care providers. For example, many raisers from both sites purchased vitamin B2 or riboflavin for their ill poultry. Some raisers purchased paracetamol, oxytetracycline, doxycycline, chlorpheniramine, calcium supplement, contraceptive pills, metronidazole and/or omeprazole for their flocks. A raiser shared,

*“During extreme heat, a virus comes from the air once in the year and poultry also defecate white lime feces like humans get diarrhea. If it happens to one chicken in a household, it spreads to other chickens of the neighboring households. Then people feed it Maya Bori, Femicon (i.e., brand names of contraceptive pills). ‘X’ (an NGO) doctors taught us this and gave our chickens this tablet. Many women don’t consume the contraceptive pills that the NGO workers distribute door-to-door themselves, rather feed their chickens when the chickens get sick. If anyone doesn’t have the pills, she borrows it from others to feed the poultry.”*

Ten (14%) of the 70 backyard poultry raisers used only home remedies. Home remedies included feeding and/or applying mustard oil, turmeric, warm rice, onion juice, juice of local herbs/leaves, soap, salt, molasses, chili powder, ginger, garlic and pain-relieving balm.

None of the 70 poultry raisers sought care from a government veterinary service provider because government livestock offices were far away and seemed to focus on large animal veterinary medicine. In contrast, mobile care providers visited the village regularly. The following quotes exemplify the preference of the raisers in both villages.

*“He (the local vendor) comes everyday... He rings the bell of his bicycle and we know that he has arrived. Then I go to him and others follow... If he doesn’t come… we go to the local market. Doctor S and M (medicine sellers at drug store) are there... They’re also human doctors. If I get fever, they give me medicine. Then if I say that my chicken is sick, they also give medicine for my chicken.”*

*“We never take backyard chickens to the livestock office. People don’t go because of expenses. It is not feasible. It takes 100-200 taka (US$ 1.3-2.6) for transportation.”*

*“Government sends people for goats and cows only. They don’t give any service for poultry... Nobody comes for backyard poultry from government offices.”*

### Local poultry care providers’ perception about avian influenza, poultry illness and treatment

Three of six local poultry care providers did not know about avian influenza. The other three local providers considered avian influenza to be a disease common among commercial poultry but not backyard poultry. For example, one stated,

*“Bird flu hasn’t spread in this area yet... It was broadcasted in TV... It occurs in farm (commercial) chickens, not in these backyard poultry. It’s a new disease. I don’t have much awareness about this disease or its symptoms.”*

The local poultry care providers from both sites used similar treatment for poultry as they did for humans or cattle with similar signs. One local care provider said that he used cattle medicine for poultry because the pharmaceutical companies printed pictures of cattle and poultry on the container. He assumed this meant that the medicine could be used for both. The local care providers were also concerned about their reputation. As one said,

*“The things you’re asking me (about poultry disease and treatment), I can’t answer. I don’t have training in chicken illnesses... I take a risk when I give treatment to the poultry based on my training on cattle... It is a matter of ‘maan-ijjot’ (prestige/reputation) to tell people that I am a cattle doctor and I don’t have training on poultry, and so cannot give treatment to the poultry.... There should be a course on poultry.”*

Poultry raisers were observed seeking care from local poultry care providers. While a researcher interviewed a mobile local care provider, three villagers came to him requesting medicines, one for a cow, one for a duck and one for a chicken. The local care provider reported that since government veterinarians did not come to the villages, villagers called him and that he had ample ‘demand’ in the area. While interviewing another local care provider at a medicine shop in the local market, the researcher observed that the local care provider provided medicine to the customer and explained the utility of the medicine.

When the customer stated, *“My chicken is drowsy, and has loose stool and fever. Give me medicine”*, the local care provider gave the customer doxycycline and told him, *“If your chicken has fever, cold or cough, it will recover. Loose stool will also recover.”*

Local poultry care providers from both sites mentioned prescribing a range of antibiotics for a variety of signs (Table 2). Oxytetracycline is the most frequently dispensed product by all vendors. They also mentioned using paracetamol for fever and cold. Unlike Chittagong vendors, Rajshahi vendors reported that they prescribed vitamin B2 or riboflavin for a number of signs. A vendor, who lived in the study village, said,

*“Anybody who has a problem will come to my home and say, ‘I have this problem. If you know what happened, give me medicine.’ Or ‘my chicken is not eating, it’s drowsy.’ Aunties come, sisters come and sisters-in-law come.”*

**Table 2:** Drugs local poultry care providers dispensed for backyard poultry in two study villages, Bangladesh, 2009

Trade name	Generic name	Signs/diseases for which dispensed by local care providers	Recommended usage (reference)	Quotes by local care providers
Renamycin	Oxytetracycline	Cholera, lime-like defecation (whitish diarrhea), liquid defecation, bloody defecation, drowsiness, Newcastle Disease, fever, cold (i.e., a respiratory illness), duck paralysis (i.e., botulism), pneumonia, weakness, loss of appetite, pox in eyes, coughing	Salmonellosis, Colibacillosis, Infectious Coryza, Chronic Respiratory Disease (CRD) or Air Sac Disease, Fowl Cholera (*Pasteurella multocida*), Necrotic Enteritis, Coccidiosis [29]	“Whatever the problem may be, if they (i.e., raisers) come to us, we first give renamycin. Antibiotics for any disease, be it cold, cough or fever... If someone hits the chicken in its leg and it is injured, painkiller works faster with renamycin. Antibiotic is a must.” “In 80% of poultry disease, tetracycline tablet like renamycin is given.” “If a raiser tells me that a chicken has fever and the chicken is big, I suggest giving a Histacin tablet, half of a Napa tablet and one fourth of a renamycin tablet. I advise to push the three medicines down the chicken’s throat with a finger and then feed them some water.” “Tetracycline starts from 250 mg for humans. Animals and birds have to be given 500 mg from the very beginning.” “These drugs (he indicted drugs at his store) are mainly for cattle, not for poultry. However, drug companies have printed picture of cattle, poultry, all animals on the containers, which implies it can be used for all.”
Dox-A vet	Doxycycline	Cholera, drowsiness, lime-like defecation, Newcastle Disease, fever, cold, duck paralysis, sneezing, coughing, pox in the eyes and face, loose stool	Chronic respiratory disease (CRD) and mycoplasmosis [29]	
Ciprofloxacin	Ciprofloxacin	Drowsiness, lime-like defecation, loose stool,	Respiratory, gastrointestinal and urinary tract infections [29]	
Histacin	Chlorphenamine Maleate	Cholera, fever, cold	Acute inflammatory and allergic conditions, or febrile illness [30]	
Napa/ Paracetamol	Acetaminophen	Fever, cold	Fever and hyperthermia and infectious diseases [30]	
Vitamin B2/B complex	Riboflavin	Weakness, paralysis, loss of appetite, edema, decreased egg laying	Vitamin B2 deficiency diseases including curled-toe, paralyzed legs, weakness, slow growth, affected egg production, diarrhea, atrophied and flabby muscles, dry and harsh skin, marked enlargement of the sciatic and brachial nerve sheaths [48]	
Metro-vet	Metronidazole	Loose stool	Protozoan and gram negative anaerobic bacterial infection [30]	
Sandocal P	Calcium supplements	Soft, thin or missing eggshell	Calcium deficiency including soft or no shell eggs [48]	
Cosomix plus/ ESB3	Sulfachloropyridazine	Liquid defecation, bloody defecation	Coccidiosis, colibacillosis, salpingitis, paratyphoid infection, staphylococcal infections, fowl cholera, infectious coryza [30]	
A-Fenac Vet	Diclofenac Sodium	Lime defecation, pox and insect in eyes	Non-steriod Anti-inflammatory drugs (NSAIDs) [30]	
Hista vet	Pheniramine Maleate	Cold	Acute inflammatory and allergic conditions, or febrile illness [30]	
Diadin	Sulphadimidine	Sore	Coccidiosis and coryza [30]	
Cotrim vet	Cotimoxazole (Sulfamethoxazole and Trimethoprim)	Bloody defecation, lime-like defecation, liquid defecation	Coccidiosis, salmonellosis, colibacillosis [30]	

The local poultry care providers reported that they were interested in supplying services to the backyard raisers because this was an income source. A vendor said,

*“There’s more money in this business. Not everyone can raise cattle but all can raise poultry... you have to go by the public demand.”*

A local care provider shared his experience during 2007–08 when bird flu outbreaks occurred in many places of Bangladesh,

*“At that time, people used to bring drowsy chickens. I used to give tetracycline… If 1 or 2 chickens got disease among 15-20 in a household, I told the raiser to separate the sick ones and give treatment separately... I don’t know anything about its (bird flu) treatment. Veterinarians might be able to say. They (villagers) didn’t know if it was bird flu.”*

### Government veterinary service providers’ role and perception about avian influenza

The government veterinary service providers had knowledge about avian influenza based on their training or experience with prevention and control. An officer said,

*“Bird flu is responsible for the die-off in chickens during the last 2 years. Bird flu transmits to humans... and infects both chickens and ducks. If anyone touches and processes a bird-flu infected chicken without gloves, human can also be infected... Humans get fever and breathing difficulty.”*

These officials stated that there were trainings for the commercial farmers in the sub-district livestock office but not for backyard poultry raisers. The officials mentioned that they offered vaccines against *Salmonella* and infectious bursal disease for commercial poultry and visited commercial poultry farms in required. However, for backyard poultry, they only offered vaccines against duck plague, duck cholera, Newcastle and fowlpox and did not provide any other service to the raisers. They reported lack of staffing and an inadequate supply of medicines for backyard poultry. A Field Assistant said that people usually came to livestock offices for the treatment of cattle; nearby backyard poultry raisers sometimes visited his office because of poultry health problems but not the raisers who lived further away.

These officials reported that they also examined and culled poultry during bird flu outbreaks. An official shared his experience culling chickens during the 2007–2008 bird flu outbreaks.

*“We had to kill chickens the whole night. While killing 20,000 chickens at-a-stretch, my gloves and mask tore, gloves slipped in sweat and I worked without protection. I thought I would die after coming out of the farm but nothing happened to me.”*

A veterinarian at sub-district level shared,

*“If anybody informed the upazilla (sub-district) livestock office (about poultry die off during a bird flu outbreak), villagers would rebuke that person... When we used to go to the village with police and a combined force to cull all poultry within 1km, villagers used to stand guard (over their flocks to resist cullers) with knives.”*

A veterinary surgeon at sub-district level said that they visited one or two commercial farms daily for bird flu but could not go door to door to check backyard poultry or visit backyard raisers because of inadequate staffing.

*“This is an animal health center. Every union is supposed to have at least one such center but all unions do not. There are two staff; one is a VFA (Veterinary Field Assistant), who performs vaccination and provides primary treatment to the animals, and the other is a FAAI (Field Assistants in Artificial Insemination), who only performs artificial insemination. But in absence of VFA, FAAI also performs responsibilities of a VFA.”*

Government providers also mentioned several reasons for raisers’ not coming to government veterinary officials, such as cost of transportation to livestock office, end of the government-sponsored free vaccine program, lack of awareness about government veterinary services and office hours that did not match poultry raisers’ busy working hours.

## Discussion

Backyard poultry raisers preferred local poultry care providers to government service providers because local providers lived closer, were available throughout the day, made house calls, were less expensive and dispensed advice and medication upon request. These findings are consistent with a systematic review that identified factors influencing care seekers to use informal providers for human health problems because of their proximity and flexible working hours [15]. Our findings suggest that local care providers could be a more effective source of communication and a more trusted source for poultry raisers compared to government counterparts and could play a role in both raising awareness among the raisers and providing practical solutions to adopt precautionary behavior. Since these local care providers belong to the community, involving them in the communication campaigns might increase community participation, incorporate local perspective to the communication, empower the community, and result in sustainable improvement in awareness and behavior.

Concern for financial gain or loss and convenience were common drivers for both the raisers and the service providers. Raisers preferred local care providers because they wanted to save money for transportation to reach government veterinarians. Since the local care providers were part of the community, villagers could easily reach them or the local providers could visit the villagers’ households. Syhakhang et al. showed that 73% of women who visited drug sellers prioritized financial constraints over quality of the drugs in Lao PDR [16]. Repackaging medications into smaller and more affordable units, providing service on credit or accepting in-kind payments when patients do not have cash make the informal providers more affordable for the poor [15, 17]. The local care providers were motivated to serve the backyard raisers for poultry problems as well as human health problems because they recognized the potential to earn a profit by dispensing medicine, as the village doctors reported in another study in Bangladesh [17]. In contrast, government veterinarians did not reach the villagers to provide care for backyard poultry and cited inadequate resource and logistic support as the major barrier. Leonard argued that permitting the government veterinarians to engage in private, fee-for-service practice in Africa provided an incentive to veterinarians to work longer hours in the rural areas [18]. However, providing service for backyard poultry might remain less attractive to government veterinarians than providing service for cattle and commercial poultry (broiler or layer chickens), since those involve higher remuneration.

Poultry raisers’ care seeking behavior for ill birds reflects a trusting relationship with the local providers, which might have contributed to their ongoing consultation with them. Informal providers possess a greater degree of perceived accountability than the formal providers because of their proximity to patients; their experiences and track record are all noted within a community, resulting in trust [19]. This trust is evidenced when the raisers reached out to the local poultry care providers for help during 2007–08 when avian influenza outbreaks occurred in many places of Bangladesh. Interpersonal communication is more effective in situations of poor literacy and low awareness than other channels of communication [20, 21]. A study on sources of information and health beliefs related to SARS and avian influenza among Chinese communities suggested that information from trusted sources, such as family and friends, contributed more to the risk perception than information from other formal sources, such as doctors and government agencies [22].

In contrast, despite knowledge and institutional training on poultry healthcare, government service providers were often perceived as distant and unwilling to accept responsibility for their services [23]. In rural Cambodia, farmers were reluctant to have their large animals vaccinated by government veterinary service providers, even when these services were provided at low subsidized costs, but were more willing to have their animals vaccinated by Village Livestock Agents [23]. The lack of government involvement in veterinary care of backyard poultry also means the government may be unaware of poultry die-offs. A study in Nigeria indicated that 57% of respondents with knowledge of avian influenza were unwilling to inform authorities of sudden and massive deaths among their flocks because of fear of culling of birds without compensation (75%) [24]. A survey in Bangladesh showed only 3% of respondents reported unusual poultry deaths to authorities, 73% did not know how to report poultry deaths and 24% did not think reporting was important [6]. As mentioned by a government official in this study, poultry culling activities during avian influenza outbreaks in 2007–08 in Bangladesh had a negative repercussion among the poultry raisers [7]. Poorly executed compensation strategies might have further weakened poultry raisers’ trust in the government and discouraged reporting unexplained flock mortality [25]. Political science scholars have noted that residents of Bangladesh generally view their government as corrupt and primarily serving the interest of government workers rather than the general public [26]. In a nationally representative survey, two thirds of Bangladeshi residents reported paying a bribe to a government official within the prior 12 months [27]. In this low trust context, it is unsurprising that poultry raisers put little trust in government veterinarians.

It is difficult to assess appropriateness of the treatments prescribed by local poultry care providers without laboratory diagnosis. The vendors reported dispensing antibiotics indiscriminately, which might increase antibiotic resistance, a global concern for both human and animal health [28], and a motivation for calls to reduce irrational antibiotic use. Medications are available for most bacterial and protozoan poultry diseases, including fowl cholera (*Pasteurella multocida*), salmonelloses, infectious coryza and coccidiosis infection [29, 30]. Viral diseases, including avian influenza, Newcastle disease, infectious bursal diseases, and infectious bronchitis, may be fatal for poultry and there is no specific antiviral treatment [31, 32]. Vaccination and improving biosecurity are keys to the prevention and control of infections in backyard poultry [33, 34]. The Government of Bangladesh produces a limited quantity of vaccine for Marek’s disease, Newcastle disease, fowl pox, pigeon pox, fowl cholera, salmonellosis, infectious bursal disease and duck plague [35], though none of the backyard raisers interviewed in these villages reported using these vaccines. The Drug Administration authority of the Government of Bangladesh has allowed restricted use of avian influenza vaccines for commercial poultry since 2014 [36], although their use is controversial because vaccinated birds can still become infected and shed viruses with few or no clinical signs of infection [37].

Local healthcare providers have been previously identified as an important source of healthcare services and influence customers’ care seeking behavior for human illness in low income countries [38–41], including Bangladesh, particularly among rural, poor and underserved populations [15, 42]. A systematic review reported that educational interventions, including capacity-building training programs for informal healthcare providers, was the most common recommendation by the authors [15]. Lack of academic or institutional training decreases the appropriateness of local healthcare providers’ recommendations [39, 40]. Recognition of a legitimate role for these informal providers is likely to provoke resistance by government sanctioned professionals which play the role of “a guarantor of standards” but are also strongly motivated to secure profits, power and privilege [43]. National health development strategies have typically ignored the existence of informal healthcare providers [42], which might result in little support to backyard poultry raisers in resource-poor settings. As Sims argues, in a country like Bangladesh, certain factors, such as complex nature of the poultry production and marketing systems, limited veterinary capacity and low level of commitment from the raisers to country-wide elimination of virus to central government, favored persistence of virus [44]. In such a scenario, co-operation between government service providers and local care providers rather than mutual exclusion, might be a better approach [18, 42] to increase veterinary service capacity to communicate recommendations to prevent avian influenza and promote biosecurity.

This research was conducted in only two sub-districts, so it may not be representative of all backyard poultry raisers or veterinary care providers of Bangladesh. Nevertheless, since responses from our study participants are consistent with behaviors of care seekers and care providers in other Bangladeshi rural communities for poultry [45, 46] and humans [17, 41], these findings are likely applicable to other similar settings. Some government veterinary service providers were reluctant to share information and the team could not corroborate some of the assertions villagers or local care providers made. The study was conducted in 2009 and there was only one reported H5N1 case prior to the data collection of this study, hence it is possible that occurrence of the latter cases, including a fatal one, might have influenced raiser’ risk perception and care seeking practices since then. However, since we did not find substantial differences in people’s awareness on avian influenza reported in surveys conducted in 2007 [2] and 2014 [47], we assume that our conclusions remain applicable. Another qualitative study that explored biosecurity practices among small commercial chicken farms during 2011–12 showed similar reliance on these local care providers [46].

## Conclusions

For a resource-poor country like Bangladesh, the local poultry care providers play an important role in providing care to backyard poultry in villages. The credibility of these vendors is linked to their relationship with the poultry raisers and reinforced by the structure of incentives. These vendors could be a useful channel to implement health promotion interventions among backyard and small commercial poultry raisers [46] because they serve both groups and are available at the village level all over the country. Local poultry care providers might also serve as a bridge between government authority and the rural raisers, since they are the persons to whom raisers reach-out for help when their birds are sick. Developing interventions to increase the knowledge and skill of these local vendors through institutional training and integrating them in the government’s avian influenza control programs is a potentially useful approach that should be evaluated.

## Abbreviations

IQR: inter-quartile range
DVM: Doctor of Veterinary Medicine.
FAAI: Field Assistants in Artificial Insemination.
VFA: Veterinary Field Assistant.
